# Cultivating community-based participatory research (CBPR) to respond to the COVID-19 pandemic: an illustrative example of partnership and topic prioritization in the food services industry

**DOI:** 10.1186/s12889-023-16787-1

**Published:** 2023-10-06

**Authors:** Michael Hoerger, Seowoo Kim, Brenna Mossman, Sarah Alonzi, Kenneth Xu, John C. Coward, Kathleen Whalen, Elizabeth Nauman, Jonice Miller, Tracey De La Cerda, Tristen Peyser, Addison Dunn, Dana Zapolin, Dulcé Rivera, Navya Murugesan, Courtney N. Baker

**Affiliations:** 1 New Orleans Louisiana (NOLA) Pandemic Food Collaborative, Tulane University, New Orleans, LA USA; 2 Department of Psychology, Tulane University, New Orleans, LA USA; 3 Departments of Psychiatry and Medicine, Tulane University, New Orleans, LA USA; 4 Freeman School of Business, Tulane University, New Orleans, LA USA; 5 Department of Palliative Medicine and Supportive Care, University Medical Center of New Orleans, New Orleans, LA USA; 6 Louisiana Cancer Research Center, New Orleans, LA USA; 7 Department of Psychology, University of California, Los Angeles, USA; 8 Louisiana Public Health Institute, New Orleans, USA

**Keywords:** Pandemics, COVID-19, Personal protective equipment, Decision making, Mental Health, Post-Acute COVID-19 syndrome, Community-based Participatory Research

## Abstract

**Background:**

As an illustrative example of COVID-19 pandemic community-based participatory research (CBPR), we describe a community-academic partnership to prioritize future research most important to people experiencing high occupational exposure to COVID-19 – food service workers. Food service workers face key challenges surrounding (1) health and safety precautions, (2) stress and mental health, and (3) the long-term pandemic impact.

**Method:**

Using CBPR methodologies, academic scientists partnered with community stakeholders to develop the research aims, methods, and measures, and interpret and disseminate results. We conducted a survey, three focus groups, and a rapid qualitative assessment to understand the three areas of concern and prioritize future research.

**Results:**

The survey showed that food service employers mainly supported basic droplet protections (soap, hand sanitizer, gloves), rather than comprehensive airborne protections (high-quality masks, air quality monitoring, air cleaning). Food service workers faced challenging decisions surrounding isolation, quarantine, testing, masking, vaccines, and in-home transmission, described anxiety, depression, and substance use as top mental health concerns, and described long-term physical and financial concerns. Focus groups provided qualitative examples of concerns experienced by food service workers and narrowed topic prioritization. The rapid qualitative assessment identified key needs and opportunities, with help reducing in-home COVID-19 transmission identified as a top priority. COVID-19 mitigation scientists offered recommendations for reducing in-home transmission.

**Conclusions:**

The COVID-19 pandemic has forced food service workers to experience complex decisions about health and safety, stress and mental health concerns, and longer-term concerns. Challenging health decisions included attempting to avoid an airborne infectious illness when employers were mainly only concerned with droplet precautions and trying to decide protocols for testing and isolation without clear guidance, free tests, or paid sick leave. Key mental health concerns were anxiety, depression, and substance use. Longer-term challenges included Long COVID, lack of mental healthcare access, and financial instability. Food service workers suggest the need for more research aimed at reducing in-home COVID-19 transmission and supporting long-term mental health, physical health, and financial concerns. This research provides an illustrative example of how to cultivate community-based partnerships to respond to immediate and critical issues affecting populations most burdened by public health crises.

Food service workers fulfill the essential societal function of ensuring access to food. The work spans multiple settings and occupations, including grocery store workers, restaurant workers, food deliverers, and more [1]. On the frontlines of the pandemic, they have frequent interactions in close proximity to densely packed groups of people, and often without rigorous health and safety protocols. Consequently, food service workers have experienced greater viral exposure, been more likely to get COVID-19 infections and reinfections, been more likely to have adverse COVID-19 outcomes, and have had some of the highest COVID-19 death rates of any occupational group [1–11]. Food service workers were among those at greatest risk at the pandemic onset, often lost jobs and health insurance during closures and reduced hours, were often among the last eligible for vaccines, and were among the first at risk of infection and reinfection when precautions were discontinued [1, 11–18]. These concerns remain ongoing today (September 15, 2023), as U.S. national wastewater data indicate that levels are higher than during 64% of pandemic days, 1.8% of the population is actively infectious, and 843,000 Americans are getting COVID-19 each day [19, 20]. The stress of the pandemic has been hard for many [21, 22], especially food service workers [11, 12, 23–28]. Overall, food service workers and their families have faced considerable challenges related to (1) COVID-19 health and safety, (2) stress and mental health, and (3) the long-term effects of the COVID-19 pandemic. These three challenges have been particularly difficult in the culinary city of New Orleans. Specifically, New Orleans is a racially, culturally, and socioeconomically diverse city that relies on tourism and dining as major sectors of the economy and had the highest mortality rate per capita of any major U.S. city at the pandemic onset, slightly higher than New York City [29]. As an illustrative example of COVID-19 pandemic community-based participatory research (CBPR), the current investigation was designed to better understand these three domains of pandemic concerns among food service workers in New Orleans.

The current research involved developing a community-academic partnership with local food service workers and their allies and colleagues — collectively termed the “stakeholder” community — from August 2021 through February 2023. We used a combination of surveys, focus groups, and qualitative methods to identify the key pandemic concerns faced by food service workers. This multimethod, iterative approach allowed us to triangulate with an increasing focus on the key issues faced by the food service worker community. The research was designed to document concerns during the course of the pandemic, set priorities for research, programs, and policy, and inform a long-term path forward for a sustainable partnership.

## Method

### Overview

This research involved a collaborative partnership among academic scientists and community stakeholders in New Orleans (food service workers, allies, and colleagues) and was designed to reveal the key pandemic concerns faced by food service workers. The academic team directly engaged stakeholders who were active on the study team. The research centered on conducting a survey of the stakeholder population, focus groups, and a qualitative assessment. Study procedures were reviewed and approved by the Tulane University Institutional Review Board (IRB # 2021 − 910).

### Project timeline

This report closely documents the timeline of study procedures because the pandemic has been marked by uncertainty (which affects planning) and volatility with respect to case rates, mitigation approaches, and scientific understanding (which affect the concerns of the day). The academic team partnered with food service workers to develop the proposal from December 2020 through April 2021, submitting a funding proposal in May 2021 for rapid review. The proposal was revised lightly in July 2021 based on the funder’s feedback, funded in August 2021, and launched in September 2021 while much of the team was evacuated out of town for up to a month due to Hurricane Ida and an extended power outage. The project ran through February 2023, with specific dates noted for each study activity.

#### Stakeholder engagement on the study team

Stakeholders contributed comprehensively to the project. Scientists and community stakeholder representatives oversaw the research as a part of a scientific advisory board, which formed in September-October 2021 and included individuals who helped submit the proposal and additional community stakeholders identified through word of mouth, social media, and listservs. Community stakeholder engagement and input guided the development of the original project proposal that was funded and supported the research, assisted with IRB, project materials, and project design, attended research team meetings, contributed to presentations, guided interpretation of the data, helped draft documents and the current manuscript, engaged in strategic planning surrounding the long-term partnership, and disseminated information to the stakeholder community. Budget planning and time commitments were discussed with each stakeholder, who provided a letter of support to align expectations, ensure equity and transparency, and ensure fair compensation. Each stakeholder was compensated for their involvement on the scientific advisory board.

The project involved academic scientists and four types of stakeholders. Academic scientists had experience in psychology, public health, epidemiology, medicine, business, and CBPR methods. The primary stakeholder group was comprised of local food service workers, which is the immediate population of interest, directly impacted by the identified problems, and most capable of informing potential solutions. The secondary group of stakeholders was family members of food service workers, who have been indirectly impacted. Next, we included people with more ancillary knowledge of issues affecting food service workers, including food business executives (expertise in understanding employer constraints) and locals knowledgeable of schools (since many food service workers have children and noted during the grant-writing phase that school COVID-19 protocols can impact the health, safety, and ability to work for others in the family). Representatives of the stakeholder community joined the academic scientists as equal members of the scientific advisory board. Stakeholders on the scientific advisory board had the option but were not required to contribute as research participants in the project survey, focus groups, or rapid qualitative assessment. The composition of the scientific advisory board was fluid as members moved out of state or took on larger or smaller roles, with typically five academic scientists and five stakeholders highly involved.

### Survey of the stakeholder population

Survey respondents were people currently working in a New Orleans food service occupation who completed a survey about their experience dealing with the pandemic. Study data were collected at the tail end of the Omicron BA.1 surge, from February 2022 through April 2022. Participants were recruited via word of mouth, email, and social media, and the research team confirmed occupational eligibility through a conversation about their work. They completed the consent form and survey online via Qualtrics. The survey assessed health and safety precautions, the stress and mental health effects of the COVID-19 pandemic, long-term health and social impact of COVID-19, and other pandemic-related concerns. Questions asked about their workplace, their self-reported experience, and their perceptions of the issues faced by other locals in the food service community. We included perceptions of the local food service community because (a) respondents may report more accurately on controversial topics (e.g., Long COVID impairment, substance use) when focused on relevant others instead of themselves, and (b) averages of multiple informants often provide a reasonably valid picture of a particular context, even when specific individuals may overestimate or underestimate community concerns. Participants were compensated with a $25 gift card for completing the survey. Descriptive analyses (means, standard deviations, frequencies) were conducted in SPSS 27.

### Focus groups

The research team conducted three sets of focus groups from April 2022 to October 2022, a period marked by low pandemic precautions and high transmission from Omicron BA.2 (dominant from April-June) and BA.5 (dominant from July-October). Each focus group corresponded to one of the three identified problem areas affecting frontline essential food service workers and their families: COVID-19 health and safety precautions (April 2022), stress and mental health (June 2022), and the long-term impact of the pandemic (October 2022). We sought to involve stakeholders most committed to each meeting’s specific problem area, based on prior informal discussions between scientists and community members, often ascertained as community members reached out to ask about the project, attended community presentations, or asked about the survey. Focus group meetings lasted approximately one hour each and occurred at times convenient for stakeholders. The focus group meetings were facilitated by the lead investigator (MH) and held remotely via Zoom to ensure safety during the pandemic. Participants were compensated with a $100 gift card for participating in a focus group.

The first focus group topic focused on health and safety issues surrounding vaccination and other COVID-related precautions. Due to high interest in the first focus group, we split participants into two subgroups held separately, one in the evening, and one the next morning. Attendees selected which time to attend. The groups discussed difficulties (e.g., biggest challenges, difficult decision-making), successes (e.g., things that have gone well), and areas for future improvement (e.g., needed resources) in relation to COVID-19 health and safety precautions.

The second round of focus groups focused on stress and mental health during the COVID-19 pandemic. The meeting discussed challenges (e.g., negative feelings, stressors), successes (e.g., financial support, local initiatives, empathy and understanding), and areas for future improvement (e.g., access to mental healthcare, support groups) in relation to stress and mental well-being.

The third round of focus groups discussed perceptions, thoughts, or opinions on long-term impacts of the COVID-19 pandemic on food service workers. The meeting discussed long-term impacts on health (e.g., long COVID-19 symptoms, long-term health needs), financial status and career goals (e.g., challenges, goals, resources, and strategies), and other items (e.g., inflation, mistreatment, attitudes).

Each meeting was audio-recorded and transcribed for the purpose of analysis. Two members of the research team coded transcripts in Atlas.ti using thematic analysis, an iterative process that allows researchers to identify and refine themes within the data. Codes, coded transcripts, and emerging themes were reviewed by three members of the research team to ensure preliminary agreement. When there were disagreements, concepts were discussed until consensus was reached. All co-authors reviewed themes for acceptability.

### Rapid qualitative assessment

In the funding proposal, we indicated that we would end the project by conducting a rapid qualitative assessment designed to obtain quick, open-ended feedback on a key topic identified as a future research priority based on the earlier survey, focus groups, and informal community feedback. The focus and methodologic details were intentionally vague in the funding proposal, as this stage was designed to be driven by prior feedback and experience and the ongoing state of the pandemic. The assessment was conducted from December 2022 to January 2023 to maximally inform the next steps of planning for future research.

Based on the collective feedback received throughout the course of the project, stakeholders and academic scientists agreed that the assessment should focus on understanding and supporting food service workers in grappling with a key issue lingering in late 2022: reducing the in-home spread of COVID-19 when a family member or housemate tests positive. Collective feedback to that point was that public health mitigation was low but that food service workers still cared deeply about reducing in-home transmission. The rapid qualitative assessment asked participants to describe their occupation or expertise and answer five open-ended questions online via Qualtrics, taking 10–20 min total. We approached this issue bidirectionally. First, food service workers completed a rapid qualitative assessment about their experiences attempting to avoid in-home transmission, challenges, and areas of uncertainty. Specifically, they were asked to describe their biggest challenges surrounding reducing in-home transmission, tips and challenges using key COVID-19 mitigation approaches, tips and challenges communicating with others about the rationale for behavior changes, advice requested from COVID-19 mitigation professionals, and other comments. Second, a national panel of COVID-19 mitigation professionals who were colleagues of the corresponding author completed a parallel assessment advising on best practices for reducing in-home transmission and handling social interactions. They were provided a scenario of a working-class two-parent family with children ages 3, 5, and 7, and asked what they would recommend the family do if the 3-year-old tested positive. Follow-up questions varied the ages of the children to make them older (i.e., 13, 15, and 17), asked about recommendations for single-parent families, asked about recommendations for higher-income families, and any other comments.

## Results

### Survey of new orleans food service workers

#### Sociodemographics

Participants (N = 23) ranged from 19 to 58 years old (Mean [SD] = 35.35 [9.60]), with 52.2% female, 56.5% non-Latino/a white, 47.8% having a college degree, 39.1% married or living with a partner, 60.9% employed full-time in the food services industry as opposed to part-time. They worked in their current job for an average of 3.10 (SD = 3.53) years and had been in the food service industry for an average of 7.28 (SD = 3.28) years, with nearly half (47.8%) having over 10 years of experience working in a food service-related occupation.

### Workplace description

Participants experienced widespread concerns related to health and safety precautions, stress and mental health, and the long-term impact of the COVID-19 pandemic (Tables 1, 2 and 3). Regarding employer-provided health and safety benefits (Table 1), respondents indicated that most employers took droplet and surface (fomite) precautions (free soap, hand sanitizer, and gloves, 56.5-73.9%) but did not take airborne precautions (CO_2_ monitoring, HEPA filters, free high-quality masks, 4.3-21.7%). Although employers encouraged food service workers to stay home when sick (69.6%), few provided paid sick leave (21.7%) nor comprehensive health benefits (mental health, vision, dental, and health insurance, 8.7-39.1%). Job satisfaction was modest (3.66 on a 1–5 scale, Table 2), and they estimated that many co-workers were dealing with financial concerns, a history of a COVID-19 infection, mental health concerns, and Long COVID (8.0-71.3%, Table 3).

**Table 1:** Food Service Worker Survey on Workplace Health and Safety Benefits, Reported Immediately Following the BA.1 Omicron Surge

Survey Result	Statistic
Free hand sanitizer, well stocked	17 (73.9%)
Encouraged to stay home when sick	16 (69.6%)
Free soap, well stocked	15 (65.2%)
Free gloves	13 (56.5%)
HVAC (heating/air conditioning) system is well-maintained	10 (43.5%)
Health insurance	9 (39.1%)
Free cloth masks	9 (39.1%)
Free surgical masks	9 (39.1%)
Free COVID-19 testing	7 (30.4%)
Paid sick leave	5 (21.7%)
Free high-quality masks, e.g., N95, N99, N100, KN95, KF94	5 (21.7%)
Dental insurance	4 (17.4%)
Vision insurance	3 (13.0%)
Mental health services/counseling	2 (8.7%)
HEPA filters are provided in areas with many people	1 (4.3%)
CO_2_ monitor is used to assess indoor air quality	1 (4.3%)

**Table 2:** Food Service Worker Survey on Self-Reported Experience with the Pandemic, Reported Immediately Following the BA.1 Omicron Surge

Survey Result	Statistic
Vaccination status, No. (%)	
None	1 (4.3%)
Johnson & Johnson, 2 shots	1 (4.3%)
Moderna, 2 shots	2 (8.7%)
Pfizer, 2 shots	5 (21.7%)
Any combination of 3 shots	14 (60.9%)
Received a COVID-19 vaccine dose in the past 6 months, No. (%)	20 (87.0%)
All eligible members of household receiving a vaccine, No. (%)	20 (87.0%)
Anyone in household too young to receive a vaccine, No. (%)	4 (17.4%)
Perceptions of vaccine safety, 0 (unsafe) to 10 (safe), M (SD)	8.87 (1.84)
Likely or extremely likely to recommend COVID-19 vaccines to others, No. (%)	20 (87.0%)
Before vaccines, concern about getting COVID-19, No. (%)	
Not at all	1 (4.3%)
A little	0 (0.0%)
Moderately	5 (21.7%)
Very	17 (73.9%)
Present concern about getting COVID-19, No. (%)	
Not at all	4 (17.4%)
A little	12 (52.2%)
Moderately	5 (21.7%)
Very	2 (8.7%)
Extent the pandemic has negatively affected one’s mental health, No. (%)	
Not at all	0 (0.0%)
Very little	2 (8.7%)
Somewhat	10 (43.5%)
To great extent	11 (47.8%)
Job satisfaction (α = 0.89), average rating from 1 (low) to 5 (high), M (SD)	3.66 (0.89)
Coping	
COPE Emotional support, 1 (low) to 4 (high), M (SD)	3.09 (0.65)
COPE Instrumental support, 1 (low) to 4 (high), M (SD)	2.87 (0.91)
Self-medicating, 1 (low) to 4 (high), M (SD)	2.52 (1.31)
COVID-19 Stress Scale, 1 (low) to 5 (high), M (SD)	2.90 (0.76)
PROMIS Life Satisfaction, 1 (low) to 7 (high), M (SD)	5.00 (1.41)
PROMIS Meaning and Purpose, 1 (low) to 5 (high), M (SD)	3.99 (0.76)
Neuro-QoL, Satisfaction with Social Roles and Activities, 1 (low) to 5 (high), M (SD)	3.26 (1.16)
PROMIS, Sleep Disturbance, 1 (low) to 5 (high), M (SD)	2.87 (0.81)
PROMIS, Sleep-Related Impairment, 1 (low) to 5 (high), M (SD)	2.91 (1.15)
Family financial concerns resulting from the COVID-19 pandemic	
Work hours cut	11 (47.8%)
Pay rate cut	10 (43.5%)
Postponed travel	10 (43.5%)
Short-term unemployment, 1–6 months	9 (39.1%)
Postponed medical or dental care	9 (39.1%)
Took an additional job	7 (30.4%)
Switched jobs	7 (30.4%)
Extra medical bills, >$500	7 (30.4%)
Loss of health insurance	7 (30.4%)
Extra expenses for comfort items, e.g., junk food, clothing, kids toys	7 (30.4%)
Difficulty making car payments	6 (26.1%)
Moving expenses	6 (26.1%)
Extra expenses for alcohol	6 (26.1%)
Long-term unemployment, > 6 months	5 (21.7%)
Late rent or mortgage payment	5 (21.7%)
Difficulty paying for utilities	5 (21.7%)
Difficulty paying tuition or student loans	5 (21.7%)
Difficulty paying for food	5 (21.7%)
Lack of stable housing	3 (13.0%)
Major health or dental issue from delayed care	3 (13.0%)
Difficulty keeping phone service	3 (13.0%)
Difficulty paying for clothing	3 (13.0%)
Difficulty paying for medications	3 (13.0%)
Extra expenses for cigarettes	3 (13.0%)
Home eviction	2 (8.7%)
Car repossessed	2 (8.7%)
Extra travel expenses, >$500	2 (8.7%)
Temporary unemployment, < 1 month	2 (8.7%)

**Table 3:** Food Service Worker Survey on the Local Food Service Worker Community, Reported Immediately Following the BA.1 Omicron Surge

Survey Result	Statistic
Estimate, percentage of co-workers dealing with a concern, M (SD)	
Financial concerns related to the pandemic	71.3% (32.0%)
History of COVID-19	53.1% (28.6%)
Mental health concerns	46.0% (34.8%)
Long COVID	8.0% (12.8%)
Estimate, whether any co-workers experience decision fatigue by area, No. (%)	
What to do if possibly sick with COVID-19	20 (87.0%)
What to do if a family member is diagnosed with COVID-19	19 (82.6%)
How to interact with customers about showing proof of vaccination	19 (82.6%)
When to return to work after COVID-19	19 (82.6%)
What to do if a family member may have COVID-19	18 (78.3%)
What to do if diagnosed with COVID-19	17 (73.9%)
How to interact with customers who dislike vaccines	17 (73.9%)
How to interact with customers who dislike masks	16 (69.6%)
When a child should return to school after COVID-19	15 (65.2%)
How to find at-home rapid tests	15 (65.2%)
Type of mask to wear	14 (60.9%)
Whether to get vaccinated against COVID-19	14 (60.9%)
Which COVID-19 vaccine to get	14 (60.9%)
Whether to get a booster	14 (60.9%)
How to deal with family members who have different COVID-19 precautions	12 (52.2%)
What to do if their kid’s school lacks COVID-19 precautions	12 (52.2%)
What precautions to take when visiting an older family member	12 (52.2%)
Whether to wear a mask	12 (52.2%)
Whether to use at-home rapid tests	8 (34.8%)
What masks their kids should wear	7 (30.4%)
How to monitor indoor air quality	5 (21.7%)
How to manage ventilation, windows or HVAC	4 (17.4%)
How to manage air filtration with HEPA or other portable air filters	3 (13.0%)
Extent discussing mental health is stigmatized in the food services, No. (%)	
Not at all	2 (8.7%)
Very little	3 (13.0%)
Somewhat	11 (47.8%)
To great extent	7 (30.4%)
Ease of access of mental health care during the pandemic, No. (%)	
Easy	2 (8.7%)
Neutral	3 (13.0%)
Difficult	10 (43.5%)
Very difficult	8 (34.8%)
“Most pressing” COVID-19-related mental health concerns, No. (%)	
Anxiety and worry	22 (95.7%)
Depression and sadness	18 (78.3%)
Substance use	16 (69.6%)
Loneliness	11 (47.8%)
Anger	11 (47.8%)
Bereavement	6 (26.1%)
Suicidal thoughts	4 (17.4%)
Violence and abuse	4 (17.4%)
“Primary Sources” of anxiety related to the pandemic, No. (%)	
Uncertainty about when things will return to normal	19 (82.6%)
Making ends meet financially	17 (73.9%)
Getting COVID-19	17 (73.9%)
Family members getting COVID-19	15 (65.2%)
Loss of income during recommended quarantine if getting COVID-19	12 (52.2%)
Job loss	11 (47.8%)
Missing work	11 (47.8%)
Having to work regardless of having symptoms	10 (43.5%
Businesses shutting down	9 (39.1%)
Lack of guidance from institutions	8 (34.8%)
Schools closing	5 (21.7%)
Finding childcare	2 (8.7%)
Level of concern about long-term effects, 1 (not at all) to 4 (very), M (SD)	
Underemployment or reduced hours	3.22 (0.80)
Long-term unemployment	3.13 (0.87)
Long-term mental health effects	3.04 (0.77)
Business closing or going under	3.04 (0.88)
Pay rate cut, e.g., reduced tips, hourly pay, or salary	3.04 (1.11)
Loss of insurance	2.87 (0.97)
Long COVID	2.83 (0.83)
Awareness of someone personally in the food service industry dealing with a symptom or side effect > 3 months after getting COVID-19 that the participant attributed to the virus (i.e., Long COVID symptoms)	
Fatigue or overtired	15 (65.2%)
Anxiety	13 (56.5%)
Depression	12 (52.2%)
Loss of taste	11 (47.8%)
Difficulty sleeping	11 (47.8%)
Headache	9 (39.1%)
Attention difficulties	9 (39.1%)
Loss of smell	7 (30.4%)
Cough	7 (30.4%)
Joint pain	6 (26.1%)
Sick to one’s stomach	6 (26.1%)
Pain	5 (21.7%)
Difficulty breathing	5 (21.7%)
Digestive problems	4 (17.4%)
Weakened lung capacity	4 (17.4%)
Weight loss	3 (13.0%)
Chest pain	2 (8.7%)
Sweats	2 (8.7%)
Occasional fever	2 (8.7%)
Vomiting or throwing up	1 (4.3%)
Hair loss	1 (4.3%)
Memory loss	1 (4.3%)

### Health decision-making

Overall, respondents noted that the local food service worker community faced considerable burdens related to health decision making (Table 3). Respondents indicated that the food service worker community struggled with what to do if they or a family member were sick or tested positive (73.9-87.0%), how to deal with customers regarding precautions (69.6-82.6%), testing concerns (34.8-65.2%), masking (30.4-69.6%), vaccinations (60 − 9-82.6%), and reducing transmission risk within one’s family (30.4-82.6%). As shown in Table 2, food services workers were highly vaccinated (95.7%, 87.0% receiving a dose in the past 6 months). Participants’ households were highly vaccinated. They viewed vaccines as safe, would recommend them to others, and were “very” concerned about COVID-19 before vaccines were available but less so after (73.9% vs. 8.7%).

### Mental health

Participants described the pandemic as negatively affecting mental health, that mental health was stigmatized, and that mental healthcare access was very difficult. Nearly half of participants (47.8%) indicated that the pandemic had affected their mental health (Table 2). Ratings of personal coping, stress, life satisfaction, meaning and purpose, social satisfaction, and sleep quality were highly variable (Table 2). As shown in Table 3, respondents estimated that the most pressing mental health concerns among local food service workers were anxiety (95.7%), depression (78.3%), and substance use (69.6%). As well, 17.4% of respondents identified suicidal ideation as the most pressing concern among local food service workers, and another 17.4% reported violence or abuse as a pressing concern. The leading primary sources of anxiety were the uncertainty of when things would return to normal (82.6%), financial concerns (up to 73.9%), and COVID-19 infections (73.9%).

### Long-term impact

Food service workers were variable in terms of the key areas where they observed long-term consequences of the COVID-19 pandemic (Tables 2 and 3). As shown in Table 2, respondents indicated that key long-term concerns among local food service workers were underemployment/unemployment, mental health, business closures, pay cuts, insurance loss, and Long COVID (means of 2.83 to 3.22 on a 1–4 scale). Within respondents’ own families (Table 2), the most common financial concerns of food service workers and their families included hour cuts, pay cuts, postponed travel, short-term unemployment, and postponed medical and dental care (39.1-47.8%). Other standout concerns include extra medical bills surpassing $500 (30.4%), extra alcohol expenses (26.1%), long-term unemployment > 6 months (21.7%), late rent/mortgage payments (21.7%), difficulty paying utilities (21.7%), home eviction (8.7%), and car repossession (8.7%). When participants were asked whether they were personally aware of a local food service worker experiencing Long COVID symptoms, top reported concerns were fatigue, anxiety, depression, loss of taste, and difficulty sleeping (47.8–65.2% of participants were aware of someone experiencing such symptoms, Table 3).

### Focus groups

The first focus group (N = 11) was split into two subgroup meetings (n of 4 and 7) and focused on COVID-19 health and safety precautions (Table 4). Key challenges included conflicts with customers, limited business due to closures and then reduced demand, personal challenges surrounding health decision making, and a lack of workplace support. Key successes included city safety precautions, workplace safety precautions, and some of the available resources, such as unemployment benefits and community meal programs. Areas for future improvement were maintaining mandates, financial support, the dissemination of information, and improvements in benefits.

**Table 4:** Summary of Themes in Focus Group 1 on COVID-19 Health Safety and Precautions

Theme	Description	Quote
**Challenges**		
Conflict with patrons	Participants described difficulty enforcing city-wide mandates, leading to conflict with patrons that often resulted in name-calling and anger directed at food service workers.	“When the vaccine mandate was enacted, we had to check the vaccination cards, and that was really hard. People fight us, telling us how it’s just theater and stupid.” “It was difficult having to be like a covid police.” “Asking people for their vaccine cards, I’ve been called a Nazi, and a lot of name calling.” “People just needed a place to vent and a person to be angry at, and we [food service workers] were those people.”
Limited business	Participants described the limited indoor dining options and staffing challenges that took a toll on business.	“In the beginning, it was really hard for us when there had to be a six feet distance between tables. Some parts of our restaurant had barely six feet between two walls. If we could only see every other table, it severely impacts the number of heads that we can serve one night.” “I remember during omicron, some restaurants had to close because all the employees were sick, out, and tested positive.”
Personal challenges	Participants described their confusion around COVID-19 tests, vaccines, and symptoms which resulted in challenges for decision-making. They often were worried about putting family members at risk. Challenging decisions about precautions often had direct financial implications.	“As a small business owner, if we caught covid, we would have to close for two weeks which means two weeks of no pay.” “Before vaccinations, you had to choose between putting yourself at risk or not making money which was definitely challenging.” “When Omicron first started, some people were testing negative one day and then testing positive the day after. It was really hard to figure out what to do.” “When my older kids went back to school and got sick, we were not sure if they had a cold or covid. So the youngest one had to get tested several times.” “I haven’t seen my own mother in three years now. I just feel like working in a restaurant will always be too much of a risk.”
Lack of workplace support	Participants expressed frustration about lack of abiding to health policies, not feeling heard by managers or employers, and being dismissed when talking openly with their managers or employers.	“I have a daughter that’s a hostess at a restaurant. When she was having symptoms, her boss asked her not to get tested for covid.” “When I felt crappy, everybody at work just kept me there because they needed me to stay.” “It was just a bunch of not regulated, not stringent boundaries. What happened is we found out about covid at work after the fact, and then the manager would say things like oh well it’s fine. When we said we wanted to go get tested, it was a problem for him as an employer.”
**Successes**		
City safety & precautions	Participants feel supported and protected by the city-wide implementation of safety and precautions, such as masking, during the pandemic.	“100% supported the city’s mask mandate and vaccine mandates. It did make me feel safer at work, even after we understood that there would be breakthrough cases.”
Workplace safety & precautions	Participants feel supported and protected by their workplaces’ implementation of safety precautions, such as providing health insurance and requiring vaccination, during the pandemic.	“My current employer requires all employees to be vaccinated, which I appreciate. It makes me feel a little bit better about working there.” “I was very lucky that they [my employer] provided tests for us if we felt symptomatic.” “Restaurant I worked at got us health insurance. It’s really nice to work in an environment where they say to not come in if you feel sick. They’re also working on getting us paid sick days now. But I do think that’s bare minimum human decency.”
Availability of resources	Participants described that resources such as unemployment benefits or mutual aid, free school lunch, and community-based resources were helpful during the pandemic.	“In the service industry, we eventually did get some unemployment and financial help, which I thought was really good.” “I know mutual aid became a much bigger thing and I became aware of mutual aid organizations after getting laid off during the pandemic.” “I appreciate that my kids are able to go to school and get free lunch. Not having to worry about paying or packing lunch is something good that happened from covid.” “Even though I haven’t utilized it, I know that people are trying to keep community fridges and pantries full during the pandemic, and I hope that people who need them are able to access them.”
**Future Improvements**		
Maintaining Mandates	Participants would like to continue or reinstate city-wide and workplace mandates and wish to see improvements in mandating safety precautions.	“I would like to see mask mandates if there’s a future pandemic.” “Bringing back the mask and vaccine mandates is always going to be on the table.”
Financial Support	Participants describe the value of financial support and wish to continue and expand support in the future.	“Financial help is always always welcomed. Just like the stimulus really helped me through it.” “Housing should be part of the financial support too, especially in New Orleans. People are being kicked from their homes because they don’t have the money to pay rent. It’s incredibly difficult to secure your housing [during the pandemic].”
Dissemination of Information	Participants desire easier access to quality information and resources about the pandemic.	“I would definitely want to see more streamlined and more available local information. I felt like all the information was coming from a lot of places and there wasn’t just one place to go for it.”
Benefits	Essential workers express a need for benefits, such as health insurance, from their employers; a need exacerbated by the pandemic.	“I hope to figure out a way to get people health insurance.” “For many reasons, there has to be a fundamental change for the restaurant workers. I was thinking about some kind of union and a livable wage.”

The second focus group (N = 9) focused on stress and mental health concerns resulting from the pandemic (Table 5). Key challenges included emotional distress (guilt, hopelessness, and uncertainty), specific stressors especially related to their families (not being able to see family, children being sick), and mental health difficulties (substance use, anxiety, and depression). Key successes included financial support that – although limited – reduced stress, and social support from friends and family. The key area of need for improvement was access to mental healthcare.

**Table 5:** Summary of Themes in Focus Group 2 on Stress and Mental Health

Theme	Description	Quote
**Challenges**		
Emotional distress	Participants described their emotional distress during the COVID-19 pandemic, including feelings of guilt, helplessness, and uncertainty.	“One time I didn’t feel well but I had tested negative so I worked a shift because my symptoms were similar to allergies, lots of sneezing and congestion. For me, it came with a lot of guilt, thinking ‘Oh my god, I just potentially exposed 60 people and a lot of them are old.’ I felt really bad because I made a lot of money while putting 60 people at risk.” “During the pandemic, there’s this uncertainty like there’s no control over who gets covid and who doesn’t. There’s also no control over who gets vaccination and who doesn’t or who wears a mask and who doesn’t. Because you have no control over pretty much anything except yourself, it causes a lot of stress.” “Even if I was following all the rules, there were all these people who were not following the rules. So there was very little actually in my control about what was happening to me and my safety. That was probably the biggest drain on my mental health.”
Stressors	Participants described issues that were most stressful for their families, households, and schools in dealing with the pandemic, including staffing, changes in protocols, and constant trauma.	“For cafeteria workers, it was a huge stressor for everyone to adapt to enormous changes at the last minute. The cafeteria staff and the teachers had to pivot from eating in the cafeteria to eating in the classroom when covid protocols came into place. That was a whole new skill set that cafeteria staff had to learn immediately. And there was a short period of flip flopping back and forth.” “So many people have left the industry during covid, so there are a lot of people now who are being given tasks and roles that maybe they’re not necessarily prepared for. So I think that causes a lot of acute stress at the moment just trying to push the food out.” “The whole lockdown and pandemic caused trauma because we lost our loved ones and good friends. Then the hurricane hit which was like trauma on top of trauma. So there has been a lot of trauma that hasn’t been addressed or taken care of when people have to go to work just to keep on going in their days as if nothing has happened.” “There’s a lot of uncertainty among people because you don’t know what you’re going to walk with every night. You could make 60 bucks or you could make 300 bucks. It’s really hard to count on that, so I think financial stress has been a huge source of anxiety for people.”
Mental health difficulties	Most participants strongly agree that substance abuse and addiction are prevalent among food service workers. Anxiety and depression are also described as common mental health issues in the industry.	“The elephant in the room with the service industry is addiction, and that’s the number one biggest mental health issue in the industry. I’m sure we all know people that we’ve worked with who died of a drug overdose. I’m not sure how much of it is self-medication because we don’t have access to mental healthcare but it’s definitely a huge huge problem.” “I had one patient that his anxiety significantly improved after the vaccine mandates were dropped because that was one of his biggest anxiety producing things, having to do that at the door and having people fight him.” “For addiction, people use alcohol to numb after a long day a lot of times. You kind of forget about how your body hurts, aches, and pains in the drink and think you can do it again.”
**Successes**		
Financial support	Participants agreed that being financially supported helped to mitigate their stress and support their mental health.	“The fact that the pay rate has increased decently is something that has been better.” “I worked for a restaurant, and after hurricane Ida, they paid us $250 a day which helped a lot. It was a huge support because I was able to pay my bills and everything.” “I think the mutual aid that cropped up and is still happening in places was really huge and very affirming.”
Social support	Participants highlighted that the pandemic resulted in more communication, flexibility, and empathy from people.	“I would agree that the pandemic definitely helped some people because some restaurants realized that they need to take better care of their staff.” “People in general have been more understanding of you. And there has been some flexibility like mental health days.” “Communication has been a little better just on a day to day basis with people.”
**Future Needs**		
Mental healthcare	Participants express a need for access to mental health services. Participants also agree that support groups or workshops would mitigate stress and mental health issues exacerbated by the pandemic.	“If I could do it, I would love to provide healthcare that is provided through restaurants. ECM access to mental health professionals right now is extremely difficult.” “I think people would be interested in a program mixture of traditional therapy and urgent care where people could regularly meet but also pop in when they’re dealing with crises. Since telehealth is huge now, it could be helpful too.” “For people who may experience substance abuse due to stress, I was thinking that support groups could be helpful.” “It would help if the restaurants would not put a black mark on somebody who needed help in that area [substance abuse] and allow them the dignity to come back to work. I think it’s important that a person can work on something they need without being ostracized and not get their job.”

The third focus group (N = 6) focused on the long-term impact of the COVID-19 pandemic (Table 6). The key issues related to health impacts were Long COVID, reinfections, and the role of employer support. Key issues related to financial and career impacts included repercussions of the larger economy, changing career plans, extra income sources, and changes in the employer’s financial strategy.

**Table 6:** Summary of Themes in Focus Group 3 on the Long-Term Impact of COVID-19

Long Term Impact of COVID-19		
Theme	Description	Quote
**Health Impact**		
Long COVID symptoms	Participants described the Long COVID symptoms that workers in the food service industry experienced and how those impacted their lives. Most commonly discussed symptoms include breathing problems, loss of taste and smell, and weakened immune system.	“I know people who have breathing problems after getting covid that they didn’t have before. And one person actually got asthma.” “I have a friend, a server who said that she can’t taste wine anymore. She lost the flavor profile so she can taste that something is alcohol but not the kind of taste. She said she can’t pick up any nuances anymore. The idea of not being able to taste wine is deeply troubling to me. I can see that really affecting someone’s career and finances.” “I have had covid twice and since then I feel like covid weakened my immune system. I am more susceptible to being sick now.”
Reinfections	Participants notice and express concern for contracting COVID-19 more than once.	“I’ve noticed that people are getting reinfected multiple times. I worked with a young lady who got covid for the fourth time and was still coming to work.”
Role of employer support	Participants have positive experiences when supported by their employers (i.e., tip pooling, health insurance, sick days), but also describe there can be a “lack of safety net” for their health without this support.	“We decided to tip pool. We take all of our tips and put them all together, and we all get paid the same wage and have five sick days a year. The way that works for us is that if we need a sick day, we’re a part of the tip pull for that day. Then we will get paid whatever everyone else does for that day.” “I started working somewhere that had been offering the employees health insurance since the pandemic.” “We still lack health care insurance and sick days. All these mean that we don’t have some sort of safety net.”
**Financial/Career Impact**		
Repercussions of larger economic stress	Participants noticed the negative economic impacts, such as inflation and shortages of food, creating negative financial stress for those in the food service industry.	“The restaurant I worked at during the worst of covid was located in the convention center. But there was no convention. I think for people who work in certain sectors of downtown, you’re pretty reliant on the tourism industry, and it was just gone.” “The inflation and shortages of food are horrible. Now it’s like how do you make a profit?” “Working at places where other service industry people hang out, we’ve seen the effects of all of us not making any money. Service workers don’t spend at those places anymore and they were the best tippers to other service industry people. So we’ve lost a big chunk of our income from us.”
Changing career plans	Participants described changes in their career plans due to financial necessity such as returning to school for further education or finding a new position.	“The pandemic accelerated me to wanting to get out of the service industry. If it wasn’t for the pandemic, I would be comfortable making that money and doing things that I wanted to do for awhile, but when covid happened, I thought I should go to school and figure something out. This is not stable.” “I just started a new job myself, and almost every single person that I’ve spoken to in the last couple of weeks were in the process of their next career jump.”
Extra income sources	Participants described ways to diversify their income sources during the pandemic including finding a side job or taking more shifts.	“I think one of the things that people have realized in the service industry is to branch out and diversify the income streams.” “I’ve been working more, picking up more shifts.”
Changes in employer financial strategy	Participants described feeling supported by many of the financial strategies implemented by their institutions during the pandemic (i.e., tip pooling, connecting on social media).	“At my restaurant, they instituted an auto-gratuity during the pandemic. I know that there’s a lot of pros and cons but it actually makes me a better server because I’m not worried about whether I’m going to make my money.” “It seems like pooled houses work really well in terms of teamwork because all staff work together for the same amount of money.” “Pooling tips gets rid of the power dynamic between the kitchen workers and people at front of the house as well as a customer.” “I saw a restaurant on Facebook that made a post asking people to please come eat with them because they were not doing well. As a restaurant owner, I know it’s a lot of pride to put that on Facebook.”

### Rapid qualitative assessment

Food service workers (N = 7) completed a rapid qualitative assessment focused on key challenges surrounding themselves or someone in their home testing positive, and COVID-19 mitigation professionals (N = 8) provided insights into various mitigation strategies aimed at reducing the likelihood of transmission (Table 7). For food service workers, key challenges included reducing in-home COVID-19 transmission, navigating work, school, and other social interactions, using different approaches to limit the spread of COVID-19, and making informed decisions about appropriate COVID-19 precautions. Specifically, food service workers expressed concerns about balancing the financial risks of prolonged isolation with safety, sought guidance on reducing transmission both at home and in the workplace, and managing the stress and mental health challenges associated with the pandemic. The workers emphasized the need for clear guidelines and support systems to navigate these complex situations, particularly in decision-making about when to drop precautions and return to work without compromising safety.

**Table 7:** Summary of the Rapid Qualitative Assessment on COVID-19 Mitigation: Perspectives from Food Service Workers and COVID-19 Mitigation Professionals

Scenario	Theme	Examples
**Food Service Workers**		
Challenges if you or someone in your home were to test positive for COVID-19	Financial	“Navigate the financial ramifications of missing work as a service industry professional if infected”
	Household safety	“Keeping my family from becoming sick” “Not having enough non-shared rooms to properly distance at home”
	Work impact	“Employers don’t really care anymore about who’s been exposed or about making people work while they’re sick.”
	Exposure	“Ensuring that I don’t become infected and transmit the illness to others at work.”
	Mental health	“Dealing with anxiety to keep my child safe from catching COVID”
Tips and challenges about COVID-19 mitigation strategies	Guidelines	Challenges: “Hard to keep up with constantly changing guidelines from the CDC, state, city”
	Precautions	Tips: “Maintaining social distancing and reintegrating with mask use seems really beneficial even if someone tests positive.”
	Resources	Challenges: “Running out of covid tests at the testing sites was a constant pain.” Tips: “It would be great if air purifiers were used in public spaces”
	Household safety	Challenges: “It is hard to stay distant from your child who has covid. You want to protect yourself but you also don’t want your loved one to feel alone.”
	Mental health	Challenges: “The constant arguments with guests in order to get them to comply with policy was a constant stress adding factor.”
Tips and challenges about COVID-19 precaution and decision-making	Work issues	Challenges: “Last year my coworkers thanked me when I still masked after being exposed or when I was feeling sick. Now I’ve had coworkers mock me for doing so.” Challenges: “People at work catch COVID but precautions are not taken seriously to avoid spread.”
	Social interaction	Challenges: “It always throws me for a loop when I’m casually discussing what I consider to be basic human decency and someone responds with annoyance. It’s baffling and discouraging.”
	Precautions	Challenges: “Not knowing the views of other people regarding COVID and the precautions that they are taking. It’s intimidating.” Challenges: “I feel as though people have truly become laxed in how they respond to covid in the workplace and may not take the precautions we did two years ago.”
**COVID-19 Mitigation Professionals**		
In-home mitigation	Masks	Wear N95 respirators (masks) or P100/N100 elastomerics if finances permit.
	Filtration	Use HEPA filters or do-it-yourself (DIY) air cleaners called Corsi-Rosenthal boxes or SAFE air purifiers.
	Ventilation	Open windows. Use fans to blow clean air in. Use fans to blow infected air out of isolation rooms.
	Isolation	Create an isolation room at home. Family members testing negative stay outside as much as possible. The person who is ill should eat outside if possible.
Testing and Treatment	Testing	Get PCR testing if possible. Use at-home rapid-antigen tests too, or at-home loop-mediated amplification (LAMP) tests if finances permit.
	Treatment	Seek Paxlovid, monoclonal antibody treatment, or other early treatments, as guidelines recommend.
Community Involvement	Work issues	Take paid sick leave or paid time off, to the extent allowed. Look for possible remote work options to make up for financial gaps.
	Social support	Reach out to family and friends to watch children while parents work, if needed. Reach out to local community resources for help.

COVID-19 mitigation professionals recommended a multi-layered approach to reducing in-home transmission that included using high-quality masks (e.g., N95), improving ventilation by bringing in outdoor air where feasible, enhancing air cleaning through HEPA filters and do-it-yourself (DIY) homemade air cleaners, such as Corsi-Rosenthal boxes [30–35]. Corsi-Rosenthal Boxes – named for the engineers that designed them – are like HEPA filters but are lower cost and can be built with supplies at most hardware stores, such as a box fan, HVAC filters, and duct tape. The professionals also stressed the importance of testing, including PCR or rapid tests, to ensure accurate isolation for positive individuals. They suggested reaching out to friends, family, and local communities for additional support and exploring remote job opportunities in case of financial difficulties.

## Discussion

**Fig. 1:**
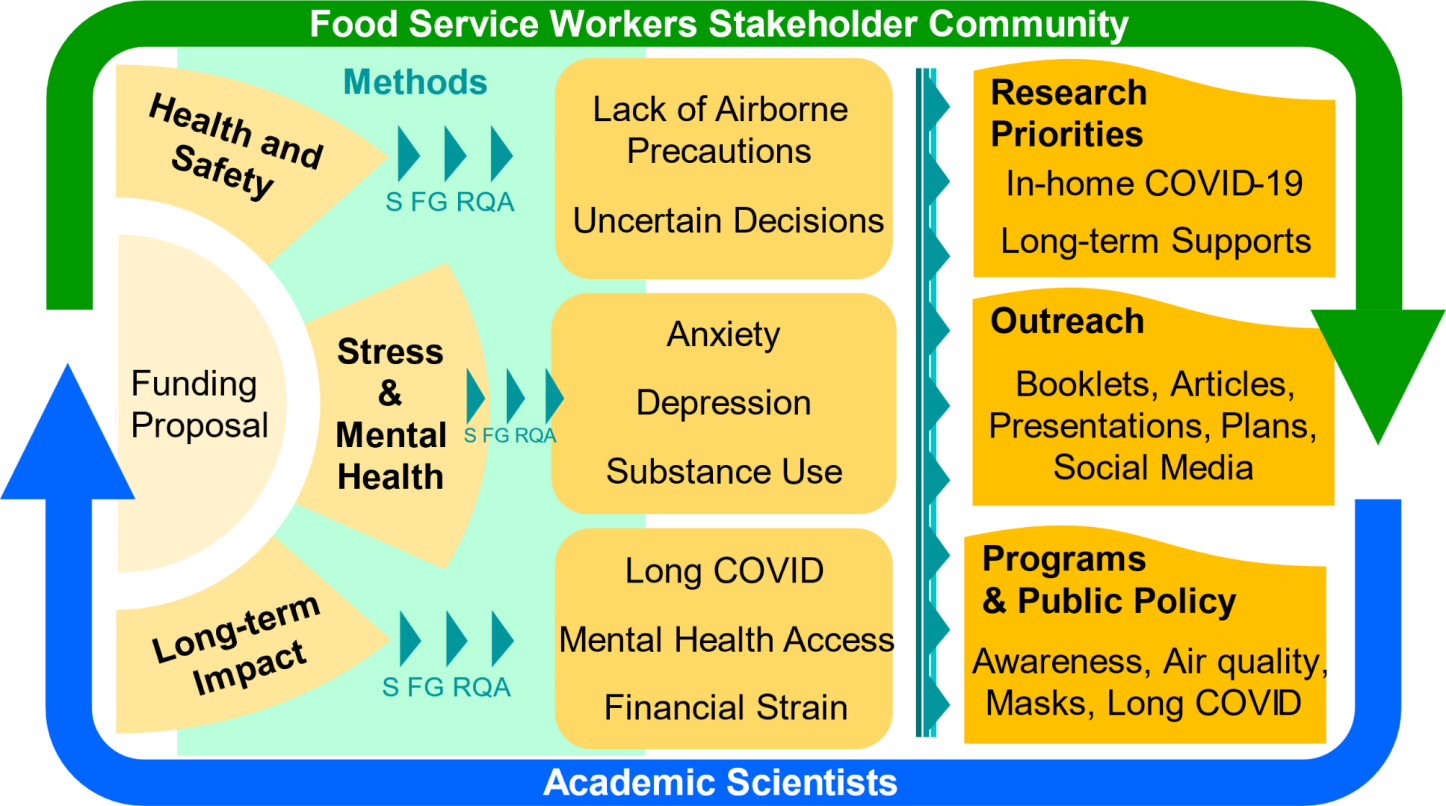
Summary of a CBPR Study Engaging the Food Service Worker Stakeholder Community and Scientists to Respond to Key COVID-19 Pandemic Concerns. The outside arrows show that community members and scientists were engaged in iterative feedback processes that encompassed all aspects of the project, from the proposal to methodology to findings to dissemination. The collaboratively designed funding proposal focused on three key issues (health and safety, stress and mental health, and long-term impact), which were studied using a combination of methods, a survey (S), three focus groups (FG), and a rapid qualitative assessment (RQA). Findings identified key concerns within each of the three topic areas. The project informed future research priorities, outreach activities, and plans for ongoing and future programs and policy initiatives

This research has documented the pandemic-related concerns of food service workers surrounding health and safety, stress and mental health, and the long-term effects of the COVID-19 pandemic. The research also provided an illustrative example of CBPR by demonstrating success in developing an academic-community partnership amid crisis. Food service workers described their experience living through the pandemic from its onset through the close of the study in February 2023, providing reasonably comprehensive coverage of the first 3 years of the pandemic from the perspective of New Orleans food service workers. Figure 1 summarizes the key findings and next steps for research, programs, and policy. Key findings were that food service workers (1) were provided workplace COVID-19 droplet-based protections that were insufficient against a highly-infectious airborne illness, (2) had to make difficult decisions about health and safety with limited definitive public health guidance and structural supports, (3) faced considerable stressors and mental health concerns, especially depression, anxiety, and substance use, with limited counseling support, (4) continue to experience long-term health, mental health, and financial impacts, and (5) want more support to prevent in-home COVID-19 transmission and gain more support around health, mental health, and financial well-being in the food service industry. Our multi-method, phased research process of moving from a survey to focus groups to a rapid qualitative assessment offered a combination of big-picture empirical evidence mixed with real-world examples and allowed us to increasingly shift from identifying problems toward targeting priorities for future solutions. Findings have implications for future research, programs, and policy aimed at mitigating the lingering impact of the COVID-19 pandemic, future pandemics and health crises, and other airborne respiratory illnesses among individuals at high risk of occupational exposure.

Although prior studies have documented some of the pandemic burdens faced by food service workers, this research highlighted the root of those burdens: food service workers were often offered low-level droplet mitigation rather than high-level airborne mitigation, creating high-exposure risk environments that led to a disproportionate burden from the pandemic. Adhering to common public health guidance, employers provided precautions mainly against basic droplet transmission (e.g., soap, hand sanitizer, gloves, low-quality masks), rather than airborne transmission (e.g., high-quality masks, ventilation, filtration via air cleaners, and air-quality monitoring). However, COVID-19 is now widely accepted to transit predominantly through the airborne route [36–40], with White House COVID-19 Response Coordinator, Ashish Jha, MD, referring to COVID-19 transmission as “purely airborne” in October 2022 [41]. Our research is the first of which we are aware to explain the pandemic-related burdens of food service work in terms of a lack of airborne COVID-19 mitigation.

This discrepancy between droplet precautions and airborne transmission helps explain prior findings that food services workers have experienced worse COVID-19 health outcomes than most other workers [1–11]. Like COVID-19, many illnesses transmit through the indoor air people breathe [40]. Recognizing the dangers of airborne illness transmission, the highest-ranking building engineering organization with 50,000 members in 130 countries, called the American Society of Heating, Refrigerating and Air-Conditioning Engineers (ASHRAE), published 2023 indoor air cleaning standards for the control of infectious aerosols [42]. The new standards indicate that restaurants and similar establishments should clean the air approximately 28–40 times per hour, depending on occupancy level (more precisely, 40 cubic feet per minute per person [cfm/person] or 20 L per second per person [l/s/person]) to reduce airborne illness transmission, approximately double the historic standard of 15 air changes per hour in U.S. operating rooms. To put in context, many restaurants, bars, and similar establishments clean the air 0.8 times per hour [43], 35–50 times lower than the current ASHRAE air cleaning standards. As the engineer Devabhaktuni Srikrishna frequently notes, even fish get 4–6 full water exchanges per hour in properly running fish tanks [44]. Essentially, food service venues are among the highest-risk settings and have the lowest mitigation. The ASHRAE standards are a firm indication of the occupational hazards of food service work. More research is needed to improve health and safety for food service workers, particularly during airborne illness crises.

Similarly, food service workers experienced a gap between what was offered and what was needed with regard to other COVID-19 health and safety concerns, stress and mental health, and the long-term impact of the COVID-19 pandemic. Regarding health and safety, employers encouraged food service workers to stay home when sick, but did not always provide free tests, guidance on testing, paid sick leave, or even health insurance. In general, food service workers faced challenging decisions surrounding vaccinations/boosters, masking, testing, isolation, quarantine, and how to reduce in-home transmission. Nonetheless, they often made wise, cautious decisions, with the vast majority having utilized vaccines, masks, testing, and routinized safety protocols. Additionally, participants indicated key concerns surrounding stress and mental health, especially related to anxiety, depression, and substance use and called for more mental health support in the community. Food service workers also indicated that they were experiencing long-term consequences of the COVID-19 pandemic in terms of mental health, Long COVID, and financial strain. Overall, food service workers were under-supported, often provided the ‘wrong’ tools or no tools at all, with broad impacts on health and mental health in the short- and long-term.

This research had strengths and limitations. The key strengths were stakeholder-engagement, community-centeredness, the use of multiple methods of assessment to triangulate priorities and capture variation over the course of the pandemic, and the innovation of responding to the pandemic in real-time, submitting a funding proposal in May 2021, when many thought the pandemic was “over,” instead of in a low point before viral evolution that produced the Delta variant, Omicron variant, and many Omicron subvariants. Limitations include the small sample sizes that are common when gathering detailed and sensitive information, the subjectivity of participants’ perspectives, and the dynamic nature of the pandemic, which means that findings at one point in time may be less relevant at a future timepoint.

Future research should focus on evaluating interventions to support the top concerns identified by stakeholders. In a world where most mitigation has been dropped, stakeholders universally cared about avoiding spreading COVID-19 within the home. At this point in time (September 2023), COVID-19 continues to transmit at a high rate, with over 800,000 American infected daily [19, 20]. In-home transmission has remained a concern throughout the pandemic [45–47]. Mitigation professionals identified actionable interventions to reduce in-home spread when someone has illness symptoms at home, including opening windows, using fans strategically, using DIY air cleaners called Corsi-Rosenthal Boxes, wearing high-quality masks, and testing to end isolation periods. Although these interventions have underlying efficacy data [30–40, 48–50], the question remains whether these specific interventions would work in the context of a comparative effectiveness trial to reduce in-home transmission under community-based circumstances with less scientific control. Such studies would be of high value for people working in settings with high transmission risk [9, 10], as well as for vulnerable populations like people with cancer or who are immunocompromised [34, 35]. Future studies should also examine interventions for reducing mental health concerns, like anxiety, depression, and substance use, as well as the financial strain exacerbated by the pandemic. This program of research would help reduce the pandemic impact experienced by people working in settings with high risk of exposure.

Although our report focuses on the development of a community-based partnership and the findings from such research, it should be noted that an important goal of CBPR is to establish long-term collaborations to drive the development of programs and policies to help the community. During the course of this partnership, we developed social media accounts, a website, and a listserv, held three public community meetings that were available live online, and wrote three brief handbooks with advice on conducting CBPR during public health crises [51–53]. These were collaborative efforts involving iterative input from scientists and the community. We have developed a strategic plan for the next three years. Moreover, we have already begun to develop programs and support improved policy initiatives for the food service worker community and others at high-exposure risk or with medical vulnerabilities [34, 35, 48, 54–56].

A few recent and ongoing examples may help illustrate how this type of project can have a broader impact on the community. Foremost, during the BA.1 Omicron surge, we led the first known research study [48] that involved distributing high-quality N95 masks to the community, launching our program before the New Orleans city and federal initiatives. We disseminated the work widely on social media, helping communities across the U.S., Canada, Europe, and Australia to develop “mask blocs” to provide free masks to those working in high-exposure settings or with medical vulnerabilities. A New Orleans mask bloc called Fight COVID NOLA – building upon but independent of our group – has now given away thousands of masks, often targeting support for the food service community. Second, we recently launched a COVID-19 dashboard [19] that uses national wastewater data to model current U.S. case rates, the percentage of the population who are actively infectious, the number of new daily Long COVID cases, and forecast future case rates. It has been viewed > 3 million times within the first 6 weeks of launch and will help people in high-risk settings to advocate for stronger mitigation. Third, in late June 2023, ASHRAE released the final draft of its standards for the control of infectious aerosols [42]. The standards use engineering terminology. We are translating that information into lay summaries and sharing through social media, recent [34, 35] and future publications, explainers, graphics, pro bono consulting with individuals and collective bargaining units, and more. As a part of our strategic plan, we will spend the next several years supporting improved air quality in restaurants, vaccine booster outreach, testing, and comprehensive interventions to reduce in-home transmission. These programs and policies will benefit the local food service worker community, food service workers in other communities, and society more broadly.

## Conclusions

In closing, this research provides an illustrative example of how to partner with stakeholders to conduct CBPR during public health crises and prioritize future research topics, programs, and policies. The top priority for future pandemic research among food service workers was to reduce in-home transmission when someone in the family tests positive for COVID-19. Moreover, the knowledge, skills, and collaborations developed through this research are expected to inform programs and policies to help food service workers and other high-exposure and vulnerable people stay safer from COVID-19.

## Data Availability

The datasets used and/or analyzed during the current study are available from the corresponding author on reasonable request.

## Abbreviations

ASHRAE: American Society of Heating, Refrigerating and Air-Conditioning Engineers
CBPR: Community-based participatory research
COVID-19: Coronavirus Disease 2019
DIY: Do it yourself
HEPA: High efficiency particulate air
SD: Standard deviation

## References

[1] Cho SJ, Lee JY, Winters JV. COVID-19 employment status impacts on food sector workers. 2020.

[2] Carlsten C, Gulati M, Hines S, Rose C, Scott K, Tarlo SM (2021). COVID-19 as an occupational disease. Am J Ind Med.

[3] Ellingson KD, Gerald JK, Sun X, Hollister J, Lutrick K, Parker J, et al. editors. Incidence of SARS-CoV-2 infection among health care personnel, first responders, and other essential workers during a prevaccination COVID-19 surge in Arizona. JAMA Health Forum; 2021: American Medical Association. 10.1001/jamahealthforum.2021.3318 PMC8727035 35977166

[4] Koh D, Goh HP (2020). Occupational health responses to COVID-19: what lessons can we learn from SARS?. J Occup Health.

[5] McClure ES, Vasudevan P, Bailey Z, Patel S, Robinson WR (2020). Racial capitalism within public health—how occupational settings drive COVID-19 disparities. Am J Epidemiol.

[6] Parks CA, Nugent NB, Fleischhacker SE, Yaroch AL (2020). Food system workers are the unexpected but under protected COVID heroes. J Nutr.

[7] Roberts JD, Dickinson KL, Koebele E, Neuberger L, Banacos N, Blanch-Hartigan D (2020). Clinicians, cooks, and cashiers: examining health equity and the COVID-19 risks to essential workers. Toxicol Ind Health.

[8] Waltenburg MA, Rose CE, Victoroff T, Butterfield M, Dillaha JA, Heinzerling A (2021). Coronavirus disease among workers in food processing, food manufacturing, and agriculture workplaces. Emerg Infect Dis.

[9] Zhang M (2021). Estimation of differential occupational risk of COVID-19 by comparing risk factors with case data by occupational group. Am J Ind Med.

[10] Billock RM, Steege AL, Miniño A. COVID-19 mortality by usual occupation and industry: 46 states and New York City, United States, 2020. 2022. 36317981

[11] Restaurant Opportunities Centers United. The Impact of COVID-19 on Restaurant Workers Across America. 2022.

[12] Bufquin D, Park J-Y, Back RM, de Souza Meira JV, Hight SK (2021). Employee work status, mental health, substance use, and career turnover intentions: an examination of restaurant employees during COVID-19. Int J Hospitality Manage.

[13] Chang S, Pierson E, Koh PW, Gerardin J, Redbird B, Grusky D (2021). Mobility network models of COVID-19 explain inequities and inform reopening. Nature.

[14] Collins C, Landivar LC, Ruppanner L, Scarborough WJ (2021). COVID-19 and the gender gap in work hours. Gend Work Organ.

[15] Dube K, Nhamo G, Chikodzi D (2021). COVID-19 cripples global restaurant and hospitality industry. Curr Issues Tourism.

[16] Asgari Mehrabadi M, Dutt N, Rahmani AM (2021). The causality inference of public interest in restaurants and bars on daily COVID-19 cases in the United States: Google Trends analysis. JMIR Public Health and Surveillance.

[17] Kawohl W, Nordt C (2020). COVID-19, unemployment, and suicide. The Lancet Psychiatry.

[18] King JS (2020). Covid-19 and the need for health care reform. N Engl J Med.

[19] Hoerger M. U.S. SARS-CoV-2 wastewater levels, COVID-19 case estimates, and 4-week forecast: Report for September 13, 2023: Pandemic Mitigation Collaborative; 2023 [Available from: http://www.pmc19.com/data.

[20] BioBot Analytics. Covid-19 Wastewater Monitoring in the U.S. 2023 [Available from: https://biobot.io/data/.

[21] Hoerger M, Alonzi S, Perry LM, Voss HM, Easwar S, Gerhart JI (2020). Impact of the COVID-19 pandemic on mental health: real-time surveillance using Google Trends. Psychol Trauma: Theory Res Pract Policy.

[22] Penninx BW, Benros ME, Klein RS, Vinkers CH (2022). How COVID-19 shaped mental health: from infection to pandemic effects. Nat Med.

[23] Adler S, Bhattacharyya S (2021). Beyond the nurses and doctors: structural racism and the unseen frontline service workers during the COVID-19 pandemic. Psychiatric Serv.

[24] Chen H, Eyoun K (2021). Do mindfulness and perceived organizational support work? Fear of COVID-19 on restaurant frontline employees’ job insecurity and emotional exhaustion. Int J Hospitality Manage.

[25] Cubrich M (2020). On the frontlines: protecting low-wage workers during COVID-19. Psychol Trauma: Theory Res Pract Policy.

[26] Lan F-Y, Suharlim C, Kales SN, Yang J (2021). Association between SARS-CoV-2 infection, exposure risk and mental health among a cohort of essential retail workers in the USA. Occup Environ Med.

[27] Rosemberg M-AS, Adams M, Polick C, Li WV, Dang J, Tsai JH-C (2021). COVID-19 and mental health of food retail, food service, and hospitality workers. J Occup Environ Hyg.

[28] Daley J. The Coronavirus Crisis: Restaurant work has become more stressful than ever. Could a staff therapist help? NPR; 2022.

[29] Calvert S. New Orleans Area Has Worst Coronavirus Death Rate in U.S. Wall Street Journal 2020 (April 4).

[30] Srikrishna D (2022). Can 10× cheaper, lower-efficiency particulate air filters and box fans complement high-efficiency Particulate Air (HEPA) purifiers to help control the COVID-19 pandemic?. Sci Total Environ.

[31] Dodson RE, Manz KE, Burks SR, Gairola R, Lee NF, Liu Y, et al. Does using Corsi–Rosenthal Boxes to mitigate COVID-19 transmission also reduce indoor air concentrations of PFAS and phthalates? Environmental Science & Technology; 2022. 10.1021/acs.est.2c05169 PMC9876422 36562547

[32] Dal Porto R, Kunz MN, Pistochini T, Corsi RL, Cappa CD (2022). Characterizing the performance of a do-it-yourself (DIY) box fan air filter. Aerosol Sci Technol.

[33] Wilke C. A conversation with Richard Corsi. ACS Publications; 2022. 10.1021/acscentsci.2c00500 PMC9136972 35647272

[34] Hoerger M, Gerhart J, Swartz MC (2023). Variability in COVID-19 vaccine response among people with cancer: what health care strategy best protects the vulnerable?. JAMA Oncol.

[35] Hoerger M, Gerhart J, Swartz MC. Evidence base for Health Care strategies to protect vulnerable patients during the COVID-19 pandemic—reply. JAMA Oncol. 2023. 10.1001/jamaoncol.2023.1520 37261807

[36] Wang CC, Prather KA, Sznitman J, Jimenez JL, Lakdawala SS, Tufekci Z (2021). Airborne transmission of respiratory viruses. Science.

[37] Greenhalgh T, Jimenez JL, Prather KA, Tufekci Z, Fisman D, Schooley R (2021). Ten scientific reasons in support of airborne transmission of SARS-CoV-2. The Lancet.

[38] Samet JM, Prather K, Benjamin G, Lakdawala S, Lowe J-M, Reingold A (2021). Airborne transmission of severe acute respiratory syndrome coronavirus 2 (SARS-CoV-2): what we know. Clin Infect Dis.

[39] Lewis D (2022). Why the WHO took two years to say COVID is airborne. Nature.

[40] Kalu IC, Henderson DK, Weber DJ, Haessler S. Back to the future: redefining universal precautions to include masking for all patient encounters. Infect Control Hosp Epidemiol. 2023:1–2. 10.1017/ice.2023.2 36762631

[41] The White House. Press Briefing by Press Secretary Karine Jean-Pierre and COVID-19 Response Coordinator Dr. Ashish Jha 2022 [Available from: https://www.whitehouse.gov/briefing-room/statements-releases/2022/10/25/press-briefing-by-press-secretary-karine-jean-pierre-and-covid-19-response-coordinator-dr-ashish-jha-6/.

[42] American Society of Heating RaA-CEA. Standard 241–2023, Control of Infectious Aerosols 2023 [Available from: https://www.ashrae.org/technical-resources/standards-and-guidelines/read-only-versions-of-ashrae-standards.

[43] Zafari Z, de Oliveira PM, Gkantonas S, Ezeh C, Muennig PA (2022). The cost-effectiveness of standalone HEPA filtration units for the prevention of airborne SARS CoV-2 transmission. Cost Eff Resource Allocation.

[44] Srikrishna D, Karan A, Dhillon RS. Making the air in the Office Cleaner. Harvard Business Rev. 2023.

[45] Lei H, Xu X, Xiao S, Wu X, Shu Y (2020). Household transmission of COVID-19-a systematic review and meta-analysis. J Infect.

[46] Lewis NM, Chu VT, Ye D, Conners EE, Gharpure R, Laws RL (2021). Household transmission of severe acute respiratory syndrome coronavirus-2 in the United States. Clin Infect Dis.

[47] Allen H, Vusirikala A, Flannagan J, Twohig KA, Zaidi A, Chudasama D (2022). Household transmission of COVID-19 cases associated with SARS-CoV-2 delta variant (B. 1.617. 2): national case-control study. Lancet Reg Health-Europe.

[48] Moran JB, Dunn A, Kim S, Zapolin D, Rivera D, Hoerger M. Community-based N95 distribution during the COVID-19 Omicron BA. 1 surge: feasibility, 1-month utilization, and price implications. Translational Behav Med. 2023:ibad019. 10.1093/tbm/ibad019 37011032

[49] Rosella LC, Agrawal A, Gans J, Goldfarb A, Sennik S, Stein J (2022). Large-scale implementation of rapid antigen testing system for COVID-19 in workplaces. Sci Adv.

[50] Philippe C, Bar-Yam Y, Bilodeau S, Gershenson C, Raina SK, Chiou S-T et al. Mass testing to end the COVID-19 public health threat. Lancet Reg Health–Europe. 2023;25. 10.1016/j.lanepe.2022.100574 PMC9816799 36628300

[51] Hoerger M, Kim S, Mossman B, Baker C. The NOLA Pandemic Food Collaborative. From Early Collaboration to an Enduring Organization: The Sustainability Roadmap Toolkit: Tulane University; 2023 [Available from: http://www.nola19.com/roadmap.pdf.

[52] Xu K, Kim S, Dunn A, Zapolin D, Murugesan N, Baker CN et al. COVID-19 Research Guidebook: Tulane University; 2021 [Available from: http://www.nola19.com/covid.pdf.

[53] Alonzi S, Kim S, Zapolin D, Mossman B, Baker CN, Hoerger M et al. Scenario planning guidebook: An illustrative example from the COVID-19 pandemic: Tulane University; 2021 [Available from: http://www.nola19.com/scenario.pdf.

[54] Hoerger M, Alonzi S, Mossman B (2022). Scenario planning: a framework for mitigating uncertainty in implementing strategic behavioral medicine initiatives during the COVID-19 pandemic. Translational Behav Med.

[55] Hoerger M. Teaching a graduate-level Research Methodology Course with Comprehensive COVID-19 Precautions: implications for Safety Knowledge, Attitudes, Behavior, and inclusivity [pre-print]. Res Square. 2023.

[56] Hoerger M. Powered Air-Purifying Respirator (PAPR) and high-efficiency Particulate Air (HEPA) Buggies to improve COVID-19 safety for the Youngest Children: evaluation of prototypes [pre-print]. Res Square. 2023.

